# Supplementary material for: Equity in walking access to community home care facility resources for elderly with different mobility: A case study of Lianhu District, Xi’an
Source: PLoS One. 2022 Dec 14;17(12):e0277310. doi: 10.1371/journal.pone.0277310 (PMC9750010; doi:10.1371/journal.pone.0277310)
Supplement: Attachment — Submitted filename: Response to Reviewers.docx [file pone.0277310.s003.docx]

**Revision Report**

First of all, I would like to express our sincere gratitude to the reviewers for their comments. These comments are all valuable and helpful for revising and improving our manuscript, as well as the important guiding significance to our researches. We have studied comments carefully and have made correction which we hope meet with approval. Revised portions are marked in red in the revised version. The summary of corrections and the responses to the reviewer’s comments are listed below.

**Summary of the revision:**

**Section 1 (Introduction):** We have added a detailed description of the cited literature for the public service facilities and conclude with a brief description of the research process for the entire article.

**Section 2 (Materials and methods):** We have added a detailed description of the methodology for calculating the number of elderly people in the community, and the collection and analysis methods are described in accordance with the terms and conditions of the data source.

**Section 3 (Results):** First, we added detailed operations for selecting the elderly sample, including sampling locations, sampling subjects, and walking speed measurements, explaining the relationship between the findings and CLHLS results. Then we added the analysis and discussion of the results on the coverage, accessibility, and social equity performance of community home care facilities.

**Section 4 (Discussion):** We have added a discussion of the contribution of the analytical approach to the study and the contribution to the optimization of the configuration and layout of community home care facilities, and we presented some limitations, shortcomings, and future approaches of this paper.

**Section 5 (Conclusion):** There is too much content in the conclusion section, and we have placed some of the conclusions drawn from the data analysis in section 3 of the article.

**Response to reviewers (original comments by reviewers are in blue color)**

**Reviewer # 1:**

1. **Comment:** On page 5, there are “121” community home care service stations, which is not consistent with “117” in Table 1.
1. **Reply:** Thank you for your comment, which are very correct. The data inconsistency here is due to our oversight. The number of community home care facilities written in the text was initially collected, and we did the operation of deleting duplicate points, and the number of community home care facilities after deleting duplicate points was 117, and the subsequent database of community home care facilities built in ArcGIS 10.2 software in this paper also used these 117 service stations. Therefore, here we change “121” to “117”, and it will not affect the calculation result and writing content of the later paper.
2. **Comment:** In subsection 2.2.2, it does not clear how to calculate the number of elderly population in the community from the street-level aging rate.
2. **Reply:** In this manuscript, we calculate the number of elderly people in the community based on the 7th Census data (street-level resident population data, proportion of elderly people over 60 years old, and average household population), POI data for each community (geographic location and number of households), multiplying the number of households and average household population in each neighborhood to obtain the community population, and then multiplying the community population with the aging rate to obtain the community elderly population. We added the specific method of calculating the population of elderly in a community in subsection 2.2.2(Community data): “The data on the residential locations in Lianhu District used in this article were obtained from Shell (https://xa.ke.com/), a website that allows you to search the geographical location of all neighborhoods in Lianhu District for free. In this study, we obtained information about communities in Lianhu District form second-hand rental websites, searched 825 communities, and recorded the name, number of households, and address of each community. Based on the collected addresses of these 825 communities to search their geographic coordinates, a GIS database is created and these communities are marked on the GIS. The administrative map layer of Lianhu District in the GIS overlaps with the community layer, and the intersection tab operation in ArcGIS10.2 adds the street information of the communities in the community attribute table and exports the table. In the table, the average household population of each street level in Lianhu District from the 7th National Census of China published in 2020 was multiplied by the number of households in the community of the street to which it belongs, and the population of each community was obtained. Considering the existence of vacant houses and in order to make the population number calculation more accurate, we calculated the sum of the population numbers of the subdivisions in each street in Lianhu District, and compared the population numbers of each street level in Lianhu District from the data of the 7th National Census with the population numbers of each street calculated in this study to obtain a ratio. This ratio was multiplied by the population size of each community to obtain the population size of each community after correction for street-level census data. Finally, the aging rate of each street level in Lianhu District from the seventh national census data was multiplied by the population size of each community in the respective street to obtain the number of elderly people in each community, as shown in Fig 4.”.
3. **Comment:** In subsection 3.1, the authors should specify the measurement method of gait speed, which is the key to measuring the walking time?
3. **Reply:** Thank you for your comment, which is very helpful. In this paper, we used an unstructured observation method (open-ended observation) to measure the mobility of older adults. We selected 36 locations within nine streets in Lianhu District, which included community home care service stations, day care centers, elderly restaurants, senior centers, squares, and gardens. At each location, a section of sidewalk was selected as an observation interval, and the length of each observation interval was measured and the time taken by the elderly to pass through the interval was recorded to calculate the walking speed of the elderly. According to the measurement results, the walking speed of elderly with normal mobility, elderly with mild mobility restriction and elderly with severe mobility restriction were set to 1.1m/s, 0.67m/s and 0.44m/s, and the distance they walked for 15 minutes was 1000m, 600m and 400m. We have added a description of the walking speed measurement method and process for older adults to subsection 3.1(Analysis of community home care service ability): “We selected 36 locations in nine Streets of Lianhu District, including community home care service stations, day care centers, elderly restaurants, senior centers, squares, and gardens, the purpose was to ensure that the research targets were not only the elderly who went to the community home care facilities to receive services, but also included other elderly people, so that the sample was representative. At each location, a section of sidewalk was selected as an observation interval, and the length of each observation interval was measured and the time taken by the elderly to pass through the interval was recorded to calculate the walking speed of the elderly…. According to the findings of this paper and combined with the results of Chinese Longitudinal Healthy Longevity Survey(CLHLS), the proportion of elderly people with different mobility in Lianhu District was determined, 80% of elderly people in Lianhu District were with normal mobility, 16% were with mild mobility restriction, and 4% were with severe mobility restriction. According to the measurement results, the step speed of the elderly with normal mobility, the elderly with mild mobility limitation, and the elderly with severe mobility limitation were set to 1.1m/s, 0.67m/s, and 0.44m/s.”
4. **Comment:** In subsection 3.1, the author needs to further explain the sampling method to prove that the sample is representative. In addition, why not use the percentage of elderly with different mobility abilities in these obtained samples?
4. **Reply:** We recorded the time of elderly people walking through the observation interval by unstructured observation method, calculated their walking speed and classified them into three categories of elderly people. The research sites were divided into two categories, one at the community home care service stations, day care centers, elderly restaurants and senior centers in each street, and the other at squares and gardens where the elderly frequently move. The purpose was to ensure that the research population was not only the elderly who went to the community home care facilities to receive services, but also included other elderly people, so that the sample was representative. We have added this partial description to section 3.1(Analysis of community home care service ability) : “We selected 36 locations in nine Streets of Lianhu District, including community home care service stations, day care centers, elderly restaurants, senior centers, squares, and gardens, the purpose was to ensure that the research targets were not only the elderly who went to the community home care facilities to receive services, but also included other elderly people, so that the sample was representative.” In addition, the proportions of elderly with normal mobility, elderly with mild mobility restriction and elderly with severe mobility restriction in the 478 samples we obtained were 81.6%, 13.6% and 4.8%, and we did not discard our sampling results, but compared our sampling results with the CLHLS (Chinese Longitudinal Healthy Longevity Survey) data by taking the whole number of percentages. CLHLS, a survey organized by Peking University, is a high-quality microdata generally recognized by scholars at home and abroad, with a total sample size of 15,869 elderly people, which is much larger than our study and better reflects the real situation. The survey collected the health status of the elderly, and the survey results show that the percentage of semi-disabled elderly is about 16%. In the study of the utilization rate of community elderly facilities and the population using them, Dai and other scholars concluded that ‘there is a relatively high overlap between the elderly with mobility restriction and the semi-disabled elderly’. Therefore, we combined our sampling results with the CLHLS sampling results and set the percentages of elderly with normal mobility, elderly with mild mobility restriction, and elderly with severe mobility restriction at 80%, 16%, and 4%.
5. **Comment:** On page 13, what are the criteria for the coverage rate of community home care facilities to be considered to be low?
5. **Reply:** Thank you for your comment. The government in the study area has determined the community home care facility coverage standard from the perspective of the elderly as a whole, but has not determined the community home care facility coverage standard from the perspective of the elderly with mobility limitations. Therefore, we compared the coverage rates at the ward level with the data at the street level, and a street with a lower coverage rate than the ward as a whole is a street with a lower coverage rate.
6. **Comment:** On page 14, are walkability and accessibility the same concept? If yes, a unified word is recommended.
6. **Reply:** Thank you for your comment, it is very helpful. These two words belong to the same concept in this article and are uniformly revised to accessibility.
7. **Comment:** On page 20, “the location entropy is used to analyze the spatial pattern of the matching between the elderly with normal mobility and the elderly with and the resources of community home care facilities.” This sentence seems to have missed something after the second “with”.
7. **Reply:** Thank you for your comment, which helped us to avoid an error. The second “with” is “with mobility restriction”.
8. **Comment:** In section 4, the similarities and differences with previous studies should be further discussed. The object of Zhao’s study is lower-aged elderly people, which is not comparable with this paper.
8. **Reply:** Thank you for your comments, your suggestions are valuable and we agree. We now discuss the contribution to the study’s analytical approach and the contribution to the optimization of the configuration and layout of community home care facilities. For the research methods, we present our innovation of classifying elderly people based on their physiological differences into elderly with normal mobility, elderly with mild mobility restriction, and elderly with severe mobility restriction. Moreover, we consider the different extreme travel willingness of these three types of elderly people in the accessibility calculation process, which makes the accessibility calculation results more accurate. In terms of optimizing the configuration and layout of community home care facilities, our research can identify the weak areas and differences of community home care facilities based on the perspective of elderly with different mobility abilities, and provide targeted suggestions for the construction of community home care facilities. We have added these two discussions in detail in subsections 4.1(Contributions to research analysis methods): “This paper provides an analytical framework for the equity of access to community home care facilities resources for elderly with different mobility abilities. Based on the seventh national census data, community POI data, and map vector data, the coverage, accessibility, and spatial pattern of community-based home care facilities from the perspective of elderly with different mobility abilities are analyzed using GIS spatial analysis techniques, and the research system is scientific and operable. The research methods selected are the Kernel Density-type 2SFCA, the Gini coefficient method, the Lorenz curve method, and the Locational entropy method, which are commonly used in the evaluation of accessibility and equity of public service facilities. We have innovative points in other aspects. On the one hand, the traditional research on the accessibility of senior care facilities is aimed at the elderly as a whole and does not consider the influence of physiological differences of the elderly on the results of accessibility analysis. In contrast, the actual elderly policy is biased toward the disadvantaged groups among the elderly, and the user groups of the elderly facilities include the semi-disabled and disabled elderly. Therefore, this paper classifies elderly people according to their walking speed into elderly with normal mobility, elderly with mild mobility restriction and elderly with severe mobility restriction, and analyzes the equity in walking access to community home care facilities services from the perspective of these three groups of elderly people. The results of the analysis show that under the current construction of community home care facilities in Lianhu District, there is inequity in access to community home care facilities between the elderly with normal mobility and the elderly with mobility restriction. On the other hand, we consider the principle of facility sharing when calculating the accessibility. Although the walking speed of the three types of elderly people is different, they are in the same space to access the facility resources, so we calculate the accessibility of the three types of elderly people to access the community home care facilities resources at the same time under their different travel intentions, thus making the results of the equity analysis more accurate. The different extreme travel distances of the elderly reflect the variability of their access to community home care facilities resources, and also show that the reason for this variability is the walking speed of these three types of elderly people, and to reduce this variability, it is necessary to increase the density of community home care facilities, adjust the layout of facilities, and strengthen the road network construction. This study has some reference significance for the study of the equity of elderly care facilities.” and 4.2(Contribution to the optimization of the configuration and layout of community home care facilities): “In the context of the continuous deepening of aging, the configuration and layout of urban senior care facilities are facing an important test. Some elderly people in the ‘15-minute elderly care circle’ have access to community elderly care facilities that are much higher than the average water in the area, while some elderly people have little or no access to community elderly care facilities, which is caused by the dense elderly population and the unreasonable layout of facility configuration. This study can provide the following help for the optimization of the layout of community aged home facilities in urban communities. On the one hand, it can identify the areas with weak service capacity of community home care facilities and target these areas for the construction or renovation of community home care facilities to achieve the purpose of spatial matching of facility resources with the aged care needs of the elderly. On the other hand, it is possible to derive the differences in the access of elderly people with different mobility to community home care facilities resources, and adjust the layout of existing community home care facilities according to such differences, so that different elderly groups can have more equitable access to community home care facilities resources. Therefore, a foundation is laid for the research on the configuration and layout of community home care facilities based on elderly with different mobility.”.
9. **Comment:** Some limitations and future work should be provided.
9. **Reply:** Your suggestions are valuable and we agree with them. Your recommendations are valuable and we agree with them. There are some limitations and shortcomings in this study. First, due to the limitations of data collection, the geographic coordinates of the neighborhood and community aging-in-place facilities we collected differ from the actual entrances and exits; second, we did not consider the impact of community aging-in-place facilities in neighboring administrative districts on the access to facility resources for the elderly in this study area; third, we only chose Lianhu District as an example, and the analysis results obtained only represent Lianhu District and cannot cover all types of urban administrative units . In the future, the study should be expanded and the geographic coordinates of the entrances and exits of each community and community-based home care facilities should be accurately obtained. The influence of community home-care facilities in neighboring administrative units on this administrative unit should also be taken into account. We have added this part of the discussion to subsection 4.3(Limitations and future research): “There are some limitations and shortcomings in this study. First, due to the limitation of data acquisition, we found and marked down the vertical points of each community and community home care facilities with the nearest road network in ArcGIS software, assuming that these points are the import and export of each community and community home care facilities, which is different from the actual situation; second, we did not consider the influence of community home care facilities in adjacent administrative districts on the access of elderly people to facility resources in this study area; third, we only selected Lianhu District as an example, and the analysis results obtained only represent Lianhu District and cannot cover all types of urban administrative units. In the future, the scope of the study should be expanded. When obtaining the entrance and exit points of each district and community home care facilities, for districts with multiple entrances and exits, the location of community home care facilities around the district should be considered to determine the entrances and exits for seniors on foot, so that the research data can be more accurate. Consideration should also be given to the elderly’s access to the resources of community home care facilities in neighboring administrative districts. The residence of the elderly located at the borders of administrative districts may be closer to the senior care facilities in neighboring administrative districts, and according to the elderly’s willingness to travel, they are more willing to go to the community home care facilities in neighboring administrative districts, and this behavior will reduce the pressure of senior care service supply in this administrative district, but at the same time will increase the pressure of senior care service supply in another administrative district, which will have an impact on the urban This behavior will reduce the pressure on the supply of senior care services in this borough, but at the same time will increase the pressure on the supply of senior care services in another borough, which will have an impact on the configuration and layout of urban senior care facilities. Therefore, the results of this study can be compared with the results of the study after the inclusion of community home care facilities in neighboring boroughs, and the differences can provide reference for the allocation and layout of community home care facilities among urban boroughs.”

**Reviewer # 2:**

1. **Comment:** In the introduction, the author just lists the existing references, and detailed summary of the references is needed. At the same time, a brief description of the rest of the paper should be added at the end of the introduction.
1. **Reply:** Thank you for your comments, which have been very helpful to us. We have described and summarized in detail the literature on health care facilities, educational facilities, senior care facilities, green space park accessibility and equity mentioned in the introduction, we have added the summary in the second paragraph of Section 1(Introduction): “Scholars initially focused on health care facilities and educational facilities, in recent years there has been more research on senior living facilities and the more novel parkland. Research questions have gradually advanced from accessibility to equity. For the study of senior care facilities, scholars took the number of beds per capita in senior care facilities as the research index, and used the 2SFCA, Lorenz curve, and Locational entropy to conduct equity analysis, and the study emphasized the differences in the resources of senior care facilities per capita in each region [5]. For the study of educational facilities, scholars use the radius of school services and the radius of 15-minute living circle as accessibility evaluation indexes, and the results show that the road network is well developed in the urban core, the number of schools is large, the accessibility of neighborhoods near the core is high, and the accessibility of peripheral neighborhoods far from the core needs to be improved [6]. For the study of medical facilities, scholars took the distance from residential points to medical facilities as the accessibility evaluation index and used the 2SFCA for accessibility analysis, compared with the traditional method of the ratio of medical personnel to the population of residential points, the 2SFCA can show the spatial variation of the accessibility of medical facilities in the study area, and the results show that the accessibility of areas along transportation routes is higher [7]. For the study of park green space, scholars take the per capita park green space area and coverage rate as evaluation indexes, and consider the spatial distribution of user demand, so as to reflect the spatial match between park green space and users before [8]. By combing scholars’ literature, it can be concluded that the methods for analyzing the accessibility and equity of public service facilities mainly include the 2SFCA method, Lorenz curve method, locational entropy method, and the factors affecting the equity of facilities include the spatial distribution characteristics of different demand groups, the accessibility distribution characteristics of facilities and road network density.”. We have added a description of the rest of the paper in the last paragraph of Section 1(Introduction): “…, and then classifies the elderly into elderly with normal mobility, elderly with mild mobility restriction, and elderly with severe mobility restriction by investigating the walking speed of the elderly. The service capacity of the community home care facilities was analyzed according to the 15-minute walking distance of the elderly with different mobility; then the accessibility of the community home care facilities was measured using the Kernel Density-type 2SFCA based on the actual road network; then the Gini coefficient method, the Lorenz curve method, and the Locational entropy method were used to analyze the social equity performance. By using this series of methods to analyze the fairness of the layout of community home care facilities from the perspective of elderly people with different mobility, we grasp the differences in access to community home care facilities resources for elderly people with different mobility. Finally, discussions and conclusions of this study are presented based on the analysis results. In order to promote equitable access to community home care facilities resources for elderly with different mobility and provide a reference for community home care facilities construction.”.

**[5].**	Luo XR, Yue BJ, Lin AW. The research of accessibility and fairness of pension service facilities based on multiple modes of transportation——a case study of Wuhan. J Cent China Norm Univ Sci. 2018 [cited 7 Jun 2022]. Available: http://en.cnki.com.cn/Article_en/CJFDTotal-HZSZ201806019.htm

**[6].**	Ren RH, Wang YH, He ZW, Zhao WJ. Research on Spatial Accessibility of Primary School Education Resources in Poverty Area Based On Modified Two-step Floating Catchment Area Method ——A Case Study Of QianJiang. Geomat World. 2014 [cited 16 Sep 2022]. Available: http://en.cnki.com.cn/Article_en/CJFDTOTAL-CHRK201402006.htm

**[7].**	Hu RS, Dong SC, Hu H. A two-step Floating Catchment Area (2SFCA) Method for Measuring Spatial Accessibility to Primary Healthcare Searvice in China:A Case Study of Donghai County in Jiangsu Province. Prog Geogr. 2012;31: 1600–1607. doi:10.11820/dlkxjz.2012.12.005

**[8].**	Tan CD, Tang YH, Wu YH. Evaluation of the Equity of Urban Park Green Space Based on Population Data Spatialization: A Case Study of a Central Area of Wuhan, China. Sensors. 2019;19: 2929-. doi:10.3390/s19132929

2. **Comment:** In the materials and methods, the authors just used the existing methods (Kernel Density-type two-step floating catchment area method; Gini coefficient method and Location entropy) to analyze the problem, and do not mention the innovation or improvement of these methods.
2. **Reply:** Thank you for your comments. The Kernel Density 2SFCA method, the Gini coefficient method, the Lorenz curve and the Location entropy method used in this paper are commonly used methods to analyze the accessibility and equity of public facilities. We did not improve these methods, but our biggest innovation is to classify the elderly according to their walking speed, that is, elderly with normal mobility, elderly with mild mobility restriction and elderly with severe mobility restriction, and the research methods used in this paper are analyzed from the perspective of these three categories of elderly. When calculating the accessibility, we considered the principle of facility sharing. These three types of elderly people are in a common space to get the resources of community home care facilities, and their willingness to travel (ultimate walking distance) is different, and the distance decay coefficient is also different. Moreover, most of the previous studies use the number of elderly beds per capita as the accessibility value of elderly facilities, while this paper studies not only elderly beds, but also community home care facilities such as dining and entertainment, therefore, we use the floor area of available elderly facilities per capita as the accessibility value. The Gini coefficient method, Lorenz curve method and Locational entropy method are also calculated and plotted based on the per capita usable elderly facility floor area, which makes the whole study more rigorous.
3. **Comment:** In the results, the statistical results of many data are listed, but each result lacks corresponding discussion and result analysis.
3. **Reply:** Thank you for your comments, they are very valuable. We have conducted an equity analysis in three areas: coverage, accessibility, and social equity performance of community home care facilities, and have obtained a lot of data. With your comments, we then analyzed and discussed the results and issues reflected in these data, which reflect the variability in access to community home care facility resources and the weak areas of facility service capacity for elderly people with different mobility abilities in Lianhu District, and also show that factors such as elderly population distribution and road network density are important factors affecting equity. We added the results of the analysis of community home facilities coverage and discussion to the second paragraph of Section 3.1(Analysis of community home care service ability): “Without taking into account the actual road network, the overall coverage rate of community home care facilities in the Lianhu District from the perspective of the elderly with normal mobility and the elderly with mild mobility restriction reached the value of 90% set by the Shaanxi provincial government, except for the elderly with severe mobility restriction, which did not reach this standard. However, the overlap of the service scope of these three categories of elderly people is greater than 50%, which is too large and causes a waste of facility resources.”, and “…, the coverage rate of community home care facilities in nine streets in Lianhu District from the perspective of the elderly with normal mobility is 100%, the coverage rate of facilities in all streets from the perspective of the elderly with mild mobility restriction is over 94%, and the coverage rate of facilities in all streets from the perspective of the elderly with severe mobility restriction is the highest at 99.27% and the lowest at 67.99%, with obvious differences in coverage rates. Among them, Hongmiaopo Street, Taoyuanlu Street, Huanchengxilu Street and Zaoyuan Street have the lowest coverage rate of community home care facilities. The three reasons for the large area of the streets, the small number of facility points and the dense facility points lead to the low coverage rate of facility service scope in these streets. The most obvious street with dense facility points is Huanchengxilu Street. Huanchengxilu Street has a small area, but the community home care facilities in its streets are concentrated in the central and southern parts, resulting in low service coverage in other areas.”; the results of the analysis of accessibility and discussion to the second paragraph of Section 3.2(Accessibility analysis of community home care facilities): “This indicates that the influence of the scale of facilities, the density of facility distribution, and the degree of aggregation of the elderly population makes the accessibility of settlements in some areas vary significantly from the perspective of the elderly with normal mobility, and the overall level of accessibility is low, especially in the middle of Zaoyuan Street, the north of Hongmiaopo Street, and the south of Beiyuanmen Street.”, and “These two types of elderly with mobility restriction are also affected by the scale of facilities, the density of facility distribution, and the degree of concentration of the elderly population, and the accessibility of community home care facilities varies significantly, and the overall level is low, and there are many blind service areas. At the same time, from Figure 9B and Figure 9C, we can see that the road network density in the central part of Zaoyuan Street is low, and the accessibility value of the central part of Zaoyuan Street is as high as 5.6 from the perspective of the elderly with mild mobility restriction, and the highest accessibility value is 0.4 from the perspective of the elderly with severe mobility restriction, and the difference between the extreme travel distance of these two types of elderly with mobility restriction is 200 meters, but the accessibility value is very different, which indicates that the road network density is also one of the important factors affecting the accessibility of the facilities. This indicates that the density of the road network is also an important factor affecting the accessibility of facilities.”; and the results of the analysis of equity performance and discussion to the first, second, and fourth paragraphs of Section 3.3(Social equity performance analysis of community home care facilities): “ The calculation results show that the degree of resource allocation of community home care facilities in Lianhu District is good from the perspective of the elderly with normal mobility, while the degree of resource allocation of community home care facilities is poor and polarized from the perspective of the elderly with mobility restriction. Due to the distribution of facilities, the distribution of the elderly population and the density of the road network, the overall distribution of community home care facilities in Zaoyuan Street, Hongmiaopo Street and Beiyuanmen Street is poor, which is consistent with the results of the accessibility analysis. This indicates that the planning of community home care facilities in Lianhu District does not take into account the disadvantaged group of elderly with mobility restriction, and it reflects to a certain extent that the speed of building community home care facilities in Lianhu District cannot catch up with the speed of population aging.” and “,…indicating that the distribution of community home care facility resources among the elderly with mobility restriction in Lianhu District is polarized and there is inequity in the distribution of community home care facility resources.”.
4. **Comment:** In the discussion, an analytical framework for the evaluation of the equity of the layout of community home care facilities under the perspective of mobility is provided, however, this part is basically the result of some qualitative and lacks quantitative data discussion.
4. **Reply:** Thank you for your comments, your suggestions are valuable and we agree. The first reviewer also provided comments on the discussion section, and we now discuss the contribution to the study’s analytical approach and the contribution to the optimization of the configuration and layout of community home care facilities. For the research methods, we present our innovation of classifying elderly people based on their physiological differences into elderly with normal mobility, elderly with mild mobility restriction, and elderly with severe mobility restriction. Moreover, we consider the different extreme travel willingness of these three types of elderly people in the accessibility calculation process, which makes the accessibility calculation results more accurate. In terms of optimizing the configuration and layout of community home care facilities, our research can identify the weak areas and differences of community home care facilities based on the perspective of elderly with different mobility abilities, and provide targeted suggestions for the construction of community home care facilities. We have added these two discussions in detail in subsections 4.1(Contributions to research analysis methods): “This paper provides an analytical framework for the equity of access to community home care facilities resources for elderly with different mobility abilities. Based on the seventh national census data, community POI data, and map vector data, the coverage, accessibility, and spatial pattern of community-based home care facilities from the perspective of elderly with different mobility abilities are analyzed using GIS spatial analysis techniques, and the research system is scientific and operable. The research methods selected are the Kernel Density-type 2SFCA, the Gini coefficient method, the Lorenz curve method, and the Locational entropy method, which are commonly used in the evaluation of accessibility and equity of public service facilities. We have innovative points in other aspects. On the one hand, the traditional research on the accessibility of senior care facilities is aimed at the elderly as a whole and does not consider the influence of physiological differences of the elderly on the results of accessibility analysis. In contrast, the actual elderly policy is biased toward the disadvantaged groups among the elderly, and the user groups of the elderly facilities include the semi-disabled and disabled elderly. Therefore, this paper classifies elderly people according to their walking speed into elderly with normal mobility, elderly with mild mobility restriction and elderly with severe mobility restriction, and analyzes the equity in walking access to community home care facilities services from the perspective of these three groups of elderly people. The results of the analysis show that under the current construction of community home care facilities in Lianhu District, there is inequity in access to community home care facilities between the elderly with normal mobility and the elderly with mobility restriction. On the other hand, we consider the principle of facility sharing when calculating the accessibility. Although the walking speed of the three types of elderly people is different, they are in the same space to access the facility resources, so we calculate the accessibility of the three types of elderly people to access the community home care facilities resources at the same time under their different travel intentions, thus making the results of the equity analysis more accurate. The different extreme travel distances of the elderly reflect the variability of their access to community home care facilities resources, and also show that the reason for this variability is the walking speed of these three types of elderly people, and to reduce this variability, it is necessary to increase the density of community home care facilities, adjust the layout of facilities, and strengthen the road network construction. This study has some reference significance for the study of the equity of elderly care facilities.” and 4.2(Contribution to the optimization of the configuration and layout of community home care facilities): “In the context of the continuous deepening of aging, the configuration and layout of urban senior care facilities are facing an important test. Some elderly people in the ‘15-minute elderly care circle’ have access to community elderly care facilities that are much higher than the average water in the area, while some elderly people have little or no access to community elderly care facilities, which is caused by the dense elderly population and the unreasonable layout of facility configuration. This study can provide the following help for the optimization of the layout of community aged home facilities in urban communities. On the one hand, it can identify the areas with weak service capacity of community home care facilities and target these areas for the construction or renovation of community home care facilities to achieve the purpose of spatial matching of facility resources with the aged care needs of the elderly. On the other hand, it is possible to derive the differences in the access of elderly people with different mobility to community home care facilities resources, and adjust the layout of existing community home care facilities according to such differences, so that different elderly groups can have more equitable access to community home care facilities resources. Therefore, a foundation is laid for the research on the configuration and layout of community home care facilities based on elderly with different mobility.”.
5. **Comment:** In the conclusion, this paper constructs an evaluation framework for the equity of the layout of community home care facilities, however, the conclusion part is too long, some descriptions can be put into other modules in the paper.
5. **Reply:** Your suggestions are valuable and we strongly agree with them. In the conclusion section, we conclude that the spatial match between elderly people with mobility restriction and community home care service facilities varies greatly, and the road network density is an important factor affecting the accessibility of community home care service facilities, which is described in more detail. Therefore, we put the findings of the conclusion section in Section 3 of the article for specific description and briefly introduce these two results in the conclusion section.
